# A Case Study of Factors That Affect Secondary School Mathematics Achievement: Teacher-Parent Support, Stress Levels, and Students’ Well-Being

**DOI:** 10.3390/ijerph192316247

**Published:** 2022-12-05

**Authors:** Tommy Tanu Wijaya, Imam Fitri Rahmadi, Siti Chotimah, Jailani Jailani, Dhoriva Urwatul Wutsqa

**Affiliations:** 1 School of Mathematical Sciences, Beijing Normal University, Beijing 100875, China; 2 Linz School of Education, Johannes Kepler University Linz, 4040 Linz, Austria; 3 Fakultas Matematika dan Ilmu Pengetahuan Alam, Kampus Karangmalang, Universitas Negeri Yogyakarta, Yogyakarta 55281, Indonesia

**Keywords:** student stress, mathematics performance, learning interest, teachers-parents support

## Abstract

Psychology is one of the numerous factors that influences students’ mathematics achievement, but studies on the influence of psychology on student mathematics achievement are still limited. This study analyzes key factors affecting mathematics achievement through teacher-parent support, stress, and students’ well-being in learning mathematics. Data was collected via online questionnaires. Participants of the study are 531 students studying at five secondary schools in Bandung, Indonesia. The data were analyzed using the structural equations modeling approach using SMART-PLS 3.0 software. The results showed that interest in learning was the most significant factor affecting students’ mathematics achievement. Moreover, teachers have a more substantial effect than parents’ support, which does not significantly reduce the students’ stress levels. The academic and emotional support of teachers and parents reduces students’ stress levels while increasing their feelings and interest in learning mathematics. This study provides essential results for school teachers and parents to improve students’ mathematics achievement at the secondary school level.

## 1. Introduction

Students’ achievement is defined by the extent to which predetermined learning goals are obtained, and it is usually measured through test scores and ongoing assessments [1]. Several preliminary studies used the Grade Point Average (GPA) to analyze students’ academic achievements [2,3], while this study interpreted it as an indicator of the knowledge and understanding level of the mathematics material. It is a complex score influenced by learning media, environment, teaching methods, parental support, and personal factors [4,5]. The learning approach teachers use toward mathematics achievement has also been explored [6,7], along with the relationship between parenting style and students’ achievement [8,9]. Most studies only used a simple linear relationship to analyze its effect on students’ achievement [10,11]. Meanwhile, this study developed a new model from a psychological perspective to analyze factors strongly related to students’ mathematics achievements by adding predictors of well-being and stress levels.

Psychological factors that influence students’ mathematics achievements emotionally and academically are supported from parents and teachers. These factors definitely affect their well-being [12,13], interest in learning [14,15], and mathematics achievement [16]. The way teachers and parents support the students is a psychological construct that represents their standard strategies for teaching children [17,18]. This support is a phenomenon that is recognized and analyzed professionally to determine its effect on the students’ positive and negative behavior, subjective well-being, and learning achievement. Unfortunately, parents and teachers in Indonesia are unaware of the importance of providing academic assistance to students [19,20], hence, the majority depend more on learning models [21,22].

Mathematics mastery, inseparable from everyday activities, plays an essential role in human life [23]. However, its achievements in Indonesia are still far from expectations, as Indonesia is ranked 63 out of 70 countries according to the 2015 PISA [24]. The situation is even more worrying when students are afraid of this subject with the idea that it is difficult [25]. Therefore, the Indonesian government implemented numerous strategies to increase students’ interest in learning mathematics and acquire more achievements [22,26]. These include increasing technology-based learning media and teachers training to improve pedagogical and technological skills [27,28]. A few program extension plans have also been implemented to encourage teachers and parents to provide emotional and academic support [29,30]. Therefore, this study aims to investigate the predictors that affect students’ mathematics achievements from a psychological perspective. It also examines the predictors of parents and teachers support for students’ well-being, interest in learning mathematics, stress levels, and achievements.

Finally, this research has the expectations to contribute to providing theoretical and practical implications. Theoretically, this study will help increase knowledge and literature on research related to student mathematics achievement, especially from the aspects of parent and teacher support, stress levels, and student well-being. Practically, the results of this study can be used by teachers and parents to improve student mathematics achievement at the secondary school level.

## 2. Literature Review

This section discusses various theories underlying the study and the formulation of proposed hypotheses. It starts with an elaboration of teacher-parent support in academic and emotional matters, followed by an explanation of stress and well-being theory related to mathematics learning.

### 2.1. Teacher-Parent Support Model

Empirical studies have examined the relationship between teacher and parent support and student well-being. According to preliminary studies, teacher and parent support have numerous benefits that significantly affect student achievement and emotions [29,31,32]. Meanwhile, limited studies have combined their support regarding student stress, interest in learning, and achievement. This study showed that regardless of where the support comes from, it will always positively affect overall student well-being. However, a model is needed to determine students’ well-being, interest in learning, and achievement at the secondary school level.

### 2.2. Teachers’ Academic and Emotional Support

Several existing studies show that teachers’ support for students has a high relationship with psychological well-being. Ma et al. also stated that teachers’ support can foster student academic achievement and enjoyment [33]. Abdullah et al. reported that during the pandemic, teachers’ emotional and academic supports have a significant determination on the learning performances of undergraduate students [18]. Therefore, it can be concluded that teachers’ academic support also plays an important role in student emotions. Most students in Indonesia stay at school from 7 a.m. to 5 p.m., where they are accompanied and supported by teachers. Therefore, implementing the role of teachers as mentors to assist students academically and emotionally through fair treatment and provision of rewards helps to increase their well-being and reduce stress. It is important to investigate the novel relationship between teachers’ academic and emotional support of whether or not determine mathematics learning achievements.

### 2.3. Parents’ Support

Studies on parents’ support generally analyze the relationship between parents and students’ psychological well-being [34,35]. A study by Geng et al. (2022) and Yuill and Martin (2016) found that parental support, directly and indirectly, affects students’ physical health. Mata [29,36] stated that parental support for students at K-9 levels significantly affected their motivation and achievement. The interview results with low-socioeconomic-status children illustrated that support from parents is essential [8]. In detail, students’ physical changes can be explained by the amount of support provided by the parents. This implies that physical complaints increase in children who lack parental support and vice versa. On the other hand, students who lack parental support from childhood experience health problems and depression as they approach adulthood [37,38]. Similarly, several studies have been conducted on parental support and its relationship to students’ problems, perceived stress, well-being, achievement, and burnout [17].

In the context of this study, most Indonesian parents work hard to earn money, thereby leaving their children with their grandparents, older siblings, or teachers. These circumstances make parents unable to understand what children feel and need. Therefore, whether parent support has a direct effect on improving students’ well-being, decreasing stress, and increasing interest in learning mathematics and achievement needs to be examined. 

### 2.4. Stress and Well-Being and Mathematics Learning

There is increased stress for secondary school students in Indonesia due to demands from parents, schools, and teachers and achieving the best results. Moreover, students’ difficulty in carrying out school assignments, exams, task deadlines, and others, also cause stress [39,40]. Stress is the body’s response to environmental pressures or demands that can have positive or negative effects on a person (Bajaj et al., 2022; Choi Young-Jun and Hyosung, 2021) [41,42]. Some external factors of demand are friends, situations, learning environment, and people around students [18,43]. Stress is a natural feeling that helps individuals to deal with problems or challenges. Thoughts, motivations, and goals are internal factors. As a result of stress, a person responds physiologically and psychologically to various demands [44].

Most parents in Indonesia expect their children to have good mathematics achievements, while few assist. Several studies show that the higher the level of stress experienced by a person, the lower their achievement and well-being [45,46].

### 2.5. Interest in Learning Mathematics

Interest in learning plays a vital role in mathematics teaching activities [47,48]. When people feel pressured to do something, such as in the context of students learning and doing exercises to develop their mathematical knowledge, their interest increases. Interest is divided into two senses, namely situational and individual [14]. Situational interest is an affectionate response caused by environmental stimuli, such as technology-based learning media unfamiliar to students, and does not last long [49]. Individual interest arises from one’s perception and knowledge of content, which extends the response rate. Several factors have a relationship with students’ interest in learning mathematics. The first is confidence, which is the most important factor, where students should believe that the effort made is capable of improving their mathematical abilities [50,51]. The second is depression, which is a major cause of a lack of interest in learning [52,53]. The third is fear of failure, which is ineffective and causes irregularity in learning and working. The last is an unsupportive environment and a lack of facilities, which prevents students from learning efficiently. It can be concluded that many factors affect students’ interest when learning mathematics. Therefore, an empirical study is needed to prove these potential factors.

According to preliminary studies, the level of interest affects students’ learning motivation [15,54], self-efficacy [55], self-regulation, and overall outcomes [56,57]. Students need to learn and analyze the relationship between stress and teacher and parent support, especially in mathematics. This study also analyzes how interest in learning mathematics as a mediator affects students’ mathematics achievement. 

**Figure 1:**
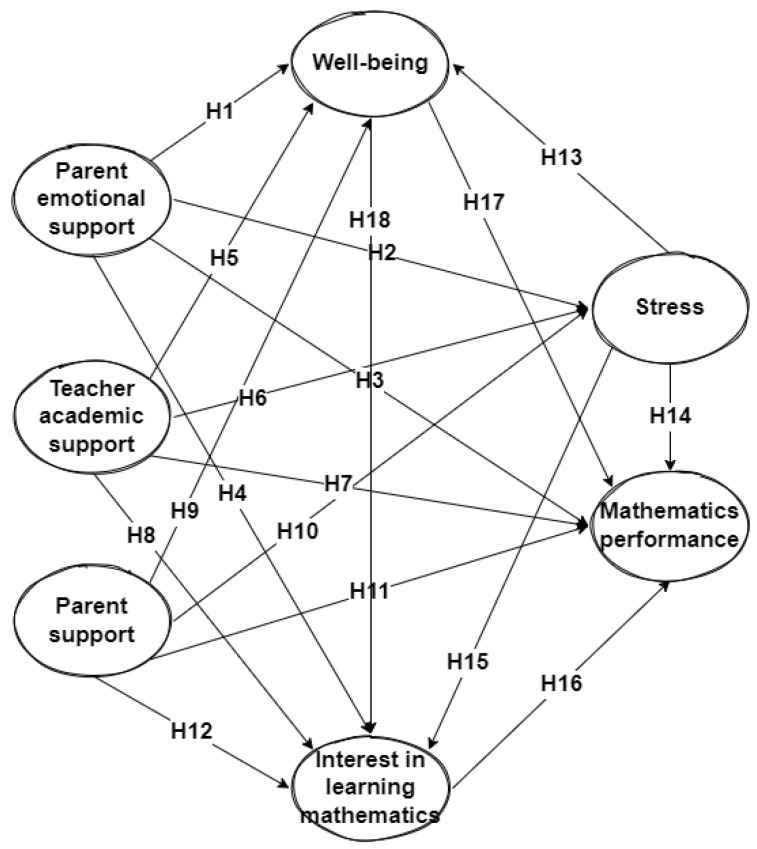
Initial hypotheses that are expected to affect students’ mathematics achievement.

The research model was constructed based on the literature review shown in Figure 1. It comprises three dependent variables, namely, parents and teachers’ academic and teachers’ emotional support. These three variables directly influence students’ well-being, interest in learning, stress, and mathematics achievement. The independent variable is students’ mathematics achievement.

### 2.6. Purpose of the Study

This study aims to determine the relationship between teacher-parent support, stress levels, and student well-being on students’ mathematics achievement. Based on the study objectives, the research hypotheses can be stated as follows:

**Hypothesis 1 (H1).**  *Parents emotional support significantly and positively affects students’ well-being.*

**Hypothesis 2 (H2).**  *Parents emotional support significantly and negatively affects students’ stress.*

**Hypothesis 3 (H3).**  *Parents emotional support significantly and positively affects students’ mathematics achievements.*

**Hypothesis 4 (H4).**  *Parents’ emotional support has a significant and positive effect on interest in learning mathematics.*

**Hypothesis 5 (H5).**  *Teachers’ academic support significantly and positively affects students’ well-being.*

**Hypothesis 6 (H6).**  *Teachers’ academic support significantly and negatively affects students’ stress.*

**Hypothesis 7 (H7).**  *Teachers’ academic support significantly and positively affects students’ mathematics achievements.*

**Hypothesis 8 (H8).**  *Teachers’ academic support significantly and positively affects an interest in learning mathematics.*

**Hypothesis 9 (H9).**  *Parents’ support has a significant and positive effect on students’ well-being.*

**Hypothesis 10 (H10).**  *Parents’ support has a significant and negative effect on students’ stress.*

**Hypothesis 11 (H11).**  *Parents’ support significantly and positively affects students’ mathematics achievements.*

**Hypothesis 12 (H12).**  *Parents’ support significantly and positively affects an interest in learning mathematics.*

**Hypothesis 13 (H13).**  *Stress has a significant and negative effect on students’ well-being.*

**Hypothesis 14 (H14).**  *Stress has a significant and negative effect on students’ mathematics achievements.*

**Hypothesis 15 (H15).**  *Stress has a significant and negative effect on interest in learning mathematics.*

**Hypothesis 16 (H16).**  *Interest in learning mathematics significantly and positively affects students’ achievements.*

**Hypothesis 17 (H17).**  *Well-being has a significant and positive effect on students’ mathematics achievements.*

**Hypothesis 18 (H18).**  *Well-being has a significant and positive effect on students’ interest in learning mathematics.*

## 3. Methodology

This research includes quantitative research, using correlational research methods with survey questionnaires. Correlational research is widely used by researchers for testing two or more variables without the researcher controlling any of them [58,59], with data collected from several secondary school students in Bandung, Indonesia, on mathematics achievement variables. Data was collected from 543 respondents from August to September 2022. After obtaining approval from the teachers at the school, an online questionnaire was given to students. Before its distribution, the contents were explained to the students, who were expected to fill it out honestly for confidential purposes without coercion. Respondent data were only used for research purposes, and students were not mandated to fill out the online questionnaire. 

After the initial analysis, only 531 respondents comprising 174 male and 357 female students completed the questionnaire with valid data for statistical processing. Of the 531 respondents, 99 were 7th grade students, while the remaining 159 and 293 were 8th and 9th grade students. Furthermore, 220 and 311 students came from private and public junior high schools, as shown in Table 1.

### 3.1. Instruments

The instrument in this study was an online questionnaire divided into two parts. The first contains the basic information about participants, and the second is associated with the questions related to factors that can affect students’ mathematics achievement at the secondary school level. The questionnaire items use a 5-point Likert scale from 1 strongly disagree to 5 strongly agree, which indicates how much students agree with the statements. The original questionnaire has seven latent variables obtained from the literature review. The latent variables include three items on interest in learning variables, 4 on mathematics learning achievement, 4 on well-being, 3 on teachers’ emotional support, 3 on teachers’ academic support, and 4 on parents’ support variables, culminating in 23 items (see Appendix A).

### 3.2. Data Analysis

The data were analyzed using SPSS 22 and smart PLS 3.0 software, which is suitable for testing hypotheses and helping new research models. Furthermore, The partial least square method structural equation model (PLS-SEM), which is a nonparametric approach, can be used to test many variables and path relationships simultaneously [60,61]. More specifically, it is used to visualize and explain the variance that exists in endogenous variables. This software is widely used to test theories that are predicted from the results of a literature review [61]. Several studies consider the PLS-SEM technique more flexible and accurate for quantifying measurement models [62,63]. This software makes it easy for respondents to be processed without considering the number of samples and the data normality and heterogeneity [64,65]. According to [66], with the PLS-SEM approach, the minimum sample should not be less than 52. In the first step, this study used SPSS software to analyze the statistical data from respondents descriptively. Descriptive statistics is a crucial step in quantitative research used to describe and summarize all respondent information in detail. Meanwhile, SMART PLS is used for data processing and distribution by looking at construct measurements, discriminant validity, and structural relationships between constructs [63]. Data reliability is also used to determine whether the questionnaire items measure the same construct. According to [67], the CR value should be greater than 0.7 to get satisfactory results. Furthermore, Ref. [68] stated that a reliable indicator’s statistical reliability value should be greater than 0.6. At the convergent validity analysis stage, external factor loadings should be greater than 0.5 with an AVE value above 0.5. Furthermore, Hair et al. [66] stated that questionnaire items are good estimators when the outer loading is greater than 0.5. Then, the HTMT value was tested to determine the correlation between constructs and analyze discriminant validity [69]. Previous studies suggested the HTMT value should not be higher than 0.9, with better performance at less than 0.85.

## 4. Results

This study was conducted to determine whether mathematics teachers’ and parents’ support affects secondary school students’ achievement and well-being. This section divides the data processing results using smart PLS software into several parts. The first descriptively analyzes the statistics of the research data and then tests the measurement model, and the last evaluates the structural model to determine the relationship between latent variables.

### 4.1. Descriptive Statistics

The descriptive statistics data in Table 2 shows that the lowest and highest items are 2.793 and 4.333, with an overall average above 3.2. Furthermore, the lowest kurtosis value of −0.910 is owned by the stress questionnaire item 2, while the highest at 1.500 is possessed by the test questionnaire item 1. According to several studies, the kurtosis and skewness values range between −7 to 7 and −2 to 2, respectively [70,71]. Table 2 also shows that the lowest and highest values of −1.024 and 0.467 are owned by items PS2 and stress 3. Therefore, all data items used in this study have acceptable skewness and kurtosis values.

### 4.2. Measurement Model Results

The first step in the measurement model analysis is to analyze the content validity. The questionnaire of this research (provided in Appendix A) was developed from the literature, and it has relatively good content validity. Furthermore, convergent validity was analyzed by evaluating the loading values, CR, AVE, and Cronbach alpha. Two questionnaire items were excluded because they have a loading factor of less than 0.7, namely, PS1 (0.69) and TAS2 (0.36). Table 3 shows a loading factor with CA and CR values more than 0.7 and AVE above 0.5 according to the recommended standard [72]. By [73] analyzing the *Rho-A* value in Table 3, it is inevitable that the construct in this study does not have a problem with composite reliability.

### 4.3. Discriminant Validity

Discriminant validity can be tested in two ways. The first is to evaluate the Fornell and Larcker values by determining the AVE square root in each latent variable [68]. The result shows that the bolded diagonal should be greater than the value of the latent variable owned by other constructs shown in Table 4. Several studies suggest that the discriminant validity test needs to be strengthened by looking at the HTMT value [74,75], which is considered to have a better benchmark. The HTMT value in Table 5 shows that all constructs are less than 0.90, which shows that the model meets the requirements of good discriminant validity.

The next stage is to check whether each item has a collinearity problem by analyzing the VIF value [76]. The recommended VIF value is less than 5 [77], and the highest obtained in this study was 3.073. Therefore, it can be ascertained that none of the items have collinearity-related problems.

### 4.4. Measurement R2 and Q2

The coefficient determination value (Table 6), commonly known as R2, is used as a reference to assess whether a model can explain an event properly [78]. The main objective of this research is to determine the effects of a mathematics teachers’ and parents’ support, stress, and well-being on students’ mathematics achievements. The determination coefficient values of 0.25, 0.5, and 0.7 are the limits that describe the quality of the model: weak, medium, and strong [79,80]. It also explains factors related to students’ mathematics achievement up to 56.4 percent. At the same time, this model can also explain 57.6 percent of the factors influencing students’ interest in learning mathematics, with a fairly strong determination coefficient.

### 4.5. Model Fit

The fit model in PLS-SEM can be analyzed from the Standardized Root Mean Square Residual (SRMR) and Normed Fit Index (NFI) values [81,82]. *SRMR* shows differences between relationships, which is considered a good fit measure in research models using PLS-SEM [62]. Values of 0.1 and 0.09 are recommended as good *SRMR*, while an *NFI* value below 1 is defined as a good fit [83]. The results of the fit model in this study can be seen in Table 7, which shows that this model has a good fit and meets the recommended fit model criteria.

### 4.6. Hypotheses Testing

**Figure 2:**
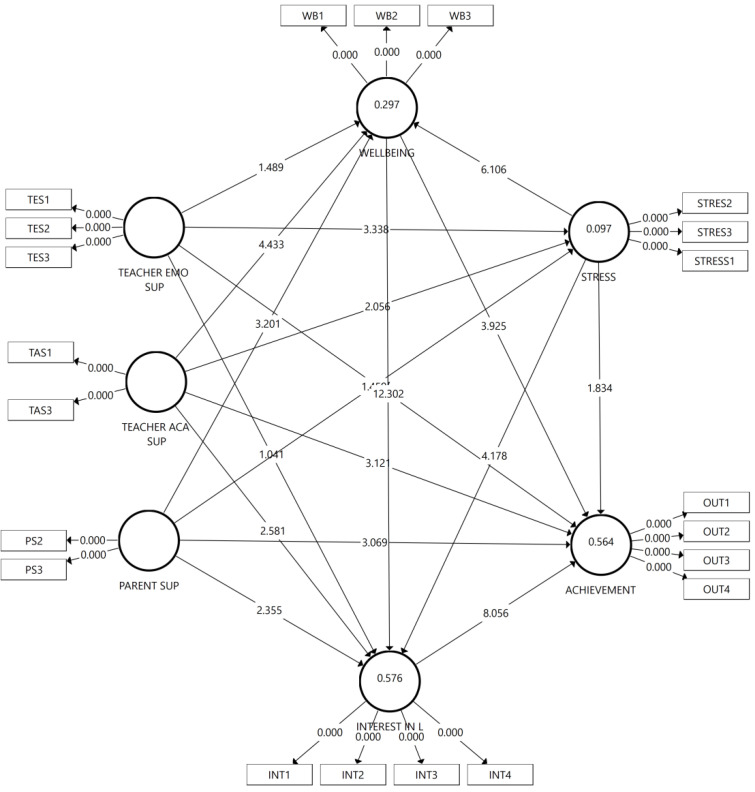
Final model with R square, *p*, and *t* values.

The 5000 resampling bootstrapping technique was used to test the hypothesis in this study [84], with the results shown in Figure 2 and Table 8. A relationship is significant if the *p* value is less than 0.05. Of the 18 initial hypotheses, 14 were supported, and 4 were unsupported. Surprisingly, teachers’ emotional support was found to have no significant relationship with students’ well-being (β = 0.076, t = 1.478, *p* = 0.140), mathematics achievement (β = −0.025, t = 0.676, *p* = 0.499), and interest (β = 0.060, t = 1.087, *p* = 0.277). However, teachers’ emotional support has a significant direct relationship to reducing stress (β = −0.182, t = 3.445, *p* = 0.001). Factors influencing students’ mathematics achievements and interest in learning are the learning media used, teaching techniques, and classroom situation. Teachers’ emotional support is not significant enough to increase students’ interest in learning mathematics and achievement. However, teachers’ emotional support is needed to reduce their stress levels while studying. This study found that stress significantly reduced well-being (β = −0.323, t = 6.156, *p* = 0.000), interest in learning (β = −0.145, t = 4.243, *p* = 0.000), and students’ mathematics achievement (β = −0.063, t = 1.858, *p* = 0.043). Teachers’ academic support in this study significantly increased well-being (β = 0.225, t = 4.519, *p* = 0.000), mathematics achievement (β = 0.132, t = 3.151, *p* = 0.002), and interest in learning (β = 0.180, t = 2.633, *p* = 0.008) with decrease in stress (β = −0.129, t = 2.066, *p* = 0.039). Parents’ support significantly affects students’ well-being (β = 0.143, t = 3.224, *p* = 0.001), mathematics achievement (β = 0.120, t = 3.111, *p* = 0.002), and interest in learning (β = 0.072, t = 2.401, *p* = 0.016), but insignificantly reduces stress (β = −0.078, t = 1.438, *p* = 0.151). Furthermore, the well-being feelings felt by students significantly affected mathematics achievement (β = 0.198, t = 4.005, *p* = 0.000) and interest (β = 0.531, t = 12.464, *p* = 0.000). Finally, interest in learning is the biggest significant factor positively affecting students’ mathematics achievement (β = 0.446, t = 4.005, *p* = 0.000).

The path model is divided into three types of effects, direct, indirect, and combined effects. According to [85], effect sizes of 0.1, 0.3, and above 0.5 are considered small, medium, and large. Table 9 shows the standardized direct, indirect, and total effects in detail, with their significance calculated using the 5000 resamplings bootstrapping technique.

The most dominant factors in increasing students’ interest in learning mathematics are their well-being during teaching activities and teachers’ academic support, with a total effect of 0.531 and 0.340. This proves that it is important to evaluate the well-being of students and teachers. Furthermore, it should be noted that the stress level students feel when learning mathematics also greatly affects their interest by −0.316.

Furthermore, teachers’ academic and emotional support significantly reduces stress levels when students learn mathematics. Finally, the factors with the highest total effect are students’ interest in learning mathematics, followed by the feeling of well-being, teachers’ academic support, and parents’ support. Their effect values are 0.446, 0.435, 0.344, and 0.235, respectively. The stress factor has an effect of −0.268 on students’ mathematics achievement.

## 5. Discussion and Implications

This study was conducted to determine the factors that psychologically influence students’ mathematics achievement. It also examines whether mathematics teachers’ and parents’ support directly influences students’ achievement and indirectly affects stress, well-being, and learning interest. The research model was developed, modified, and evaluated using empirical data from the existing teacher-parent support model [18]. These findings may help to explain the role of teachers and parents in students’ stress levels, well-being, interest, and mathematics achievement. 

The result showed that students’ well-being was not affected by teachers’ emotional support (H1) but by parents’ (H5) and teachers’ academic (H9) support. This explains why students’ feelings of well-being in Indonesia are still fully dependent on their parents. The study showed that 1 out of 3 students (39%) with low well-being rarely or never talk about their problems and possess a low level of communication to share their feelings daily. This study provides new knowledge which enables parents to change roles as friends or siblings to support their child’s well-being. It enables them to communicate positively and pay attention to their children’s problems. It also inspires teachers to support students in various activities at school and pay more attention to them, which positively affects their achievement [86,87].

Meanwhile, this study also found that parents’ support did not significantly reduce students’ stress levels when learning mathematics (H10). Students felt that academic teaching (H2) and emotional (H6) support can reduce their stress levels when learning mathematics. They felt that the stress caused by mathematics lessons is more effective when conveyed to teachers at school. This finding provides suggestions for teachers to understand that students have diverse abilities. Therefore, varying learning approaches and models are needed to support them in mathematics lessons while simultaneously reducing the stress level caused by learning it in the classroom. The use of technology-based learning media can reduce students’ stress levels. Several studies have shown that using ICT in the classroom improves students’ soft skills [88,89,90]. However, teachers may need more time to understand students’ individual characters in order to provide appropriate support.

Based on the factors related to students’ interest in learning mathematics, the study found that teacher academic support (H8), parent support (H12) and stress level factors (H15) significantly have a relationship with learning interest. Meanwhile, the emotional support provided by the teachers does not significantly affect students’ interest in learning. Several preliminary studies also found that students’ stress levels reduce interest in learning [91]. This is because those who feel stressed are usually excessively anxious, therefore, they lose their sense of interest. Moreover, secondary school students tend to avoid stress rather than tackle it, which makes the role of teachers and parents very important. Parents can support students by creating a comfortable learning atmosphere and providing adequate support learning materials within and outside the school. Meanwhile, teachers can select attractive learning media and provide easy learning methods capable of increasing students’ interest in learning mathematics [92].

The latest findings are factors related to students’ mathematics achievement, including teaching academic support (H7), parent support (H11), stress levels (H14), interest in learning (H15), and well-being (H17). However, well-being is not the main factor that significantly affects students’ mathematics achievement. This is explained by the fact that schools focus more on cognitive and academic performance. Adler stated that teaching students about well-being increases their academic achievement. These findings are appropriate to the results of a meta-analysis [93] which showed a relationship between well-being and students’ achievement, which only had a small effect. The stress level can also reduce student achievement, when increased.

This study provides several theoretical and practical implications. Firstly, it modifies and develops a research model from the teacher-parent support model by adding additional predictors and strengthening explanation power. Secondly, it provides theoretical implications for exploring the relationship between mathematics teachers’ and parents’ support on students’ stress levels, well-being, learning interest, and mathematics achievement to fulfill the conceptual framework, especially in mathematics education, for developing countries such as Indonesia. It is necessary to understand that teacher and parental support at school and home has the same effect on students’ well-being, reduces stress, and increases interest in learning. This opens up new knowledge for parents to support children both academically and emotionally.

This study provides a deeper understanding of the factors that affect students’ mathematics achievement in developing countries, especially Indonesia. The results educate parents, teachers, schools, and students about the importance of teachers’ support for students while studying mathematics. This is because it increases their well-being and reduces stress levels despite the difficulty attached to learning this subject. It also indicates that students’ mathematics achievement depends not only on the learning media and the teachers’ teaching abilities but on the used educational technologies and the drill-and-practice of mathematical tasks as well. However, it is also associated with the psychological factors, namely, stress, well-being, interest in learning, and parental support.

Therefore, there are several vital points associated with this study. Firstly, the parents’ role is essential to students’ mathematics achievement, learning interest, and stress levels. Parents should set aside time to assist their children because it increases their self-confidence and motivation to learn, improving mathematics achievement. Students do not need parents to be able to teach or answer existing mathematics material, but parental support is important. Secondly, school mathematics teachers need to understand the importance of psychological support for students [94,95]. For instance, K-12 students consider support from people in their environment very important [96,97].

Students are not usually able to independently motivate themselves, hence, they are easily stressed. Furthermore, due to their varying abilities, it would be imperative for mathematics teachers to provide academic support to benefit the emotional and educational support of those with lower abilities. Thirdly, schools can provide briefings or training for teachers and parents on the importance of maintaining students’ interest in learning and the need to encourage them academically and emotionally continuously. Students’ growth, development, and achievement depend not only on the teachers at the school but is the task of both teachers and parents.

## 6. Conclusions

In conclusion, this study was conducted to determine whether teacher and parent support are related to students’ achievement, stress levels, and interest in learning mathematics in Indonesia. Initial hypotheses were developed based on literature reviews and modification of the current teacher-parent support model. This study developed and validated a model to significantly increase students’ mathematics achievement at the secondary school level. The model provides new ideas and knowledge that are important and need to be implemented in Indonesia, significantly changing parents’ perspectives of students. Furthermore, it analyzed students’ well-being, which is essential in increasing their interest in learning mathematics and achieving success. Reducing stress levels and increasing feelings of well-being affect students’ mathematics achievement. Therefore, teachers should not only master how to teach mathematics but also need to have some knowledge of students’ psychology.

The results also showed that students in Indonesia have high enthusiasm for their parents and teachers to reduce their stress levels with an increase in overall well-being while learning mathematics. They also think that their mathematics achievement is influenced by the academic and emotional support provided by teachers and parents at home. However, more efforts are needed to improve teachers’ abilities to support students psychologically successfully. This discovery is a new step and attitude that requires more effort from parents and teachers. This study opens up initial knowledge about the importance of teacher and parent support for students’ mathematics achievement. Further studies need to be conducted to support the results of this study.

## 7. Limitations

Although this study provides several implications and contributes to the mathematics education field, it has some limitations that can be a starting point and recommendations for further research. For instance, it uses a correlational design, which makes it prone to bias when data collection is analyzed. Furthermore, it was only conducted at the secondary school level, hence, future studies need to test the developed models. Although the results need to be interpreted carefully, they cannot be generalized because the sample used in this study was less than 500 secondary school students in West Java, Indonesia. Therefore, further studies need to be conducted using a larger scale of respondents to prove the findings and determine the comparative narrative between countries.

This study also recommends the development of new research models at other education levels. Similar and different effects on other subjects need to be further investigated. Several factors related to the stress and well-being model may also be added to the research model to investigate whether they relate to students’ mathematics achievement.

## Figures and Tables

**Table 1:** Descriptive statistics of students in this study.

Measure	Items	N	Percentage
Gender	male	174	32.77%
	female	357	67.23%
Class	7	99	18.64%
	8	159	29.94%
	9	293	55.18%
School Type	Public school	311	58.57%
	Private school	220	41.43%
Total		531	100%

**Table 2:** Descriptive statistics in detail.

Item	Mean	Median	Min	Max	Standard Deviation	Kurtosis	Skewness
INT1	3.627	4.000	1.000	5.000	0.821	0.879	−0.893
INT2	3.874	4.000	1.000	5.000	0.757	1.603	−0.933
INT3	3.488	4.000	1.000	5.000	0.822	0.244	−0.502
INT4	3.505	4.000	1.000	5.000	0.789	0.407	−0.511
OUT1	3.569	4.000	1.000	5.000	0.894	−0.043	−0.485
OUT2	3.808	4.000	1.000	5.000	0.737	1.174	−0.670
OUT3	3.574	4.000	1.000	5.000	0.814	0.489	−0.483
OUT4	3.650	4.000	1.000	5.000	0.768	0.410	−0.411
WB1	3.616	4.000	1.000	5.000	0.878	0.332	−0.590
WB2	3.576	4.000	1.000	5.000	0.823	0.845	−0.804
WB3	3.245	3.000	1.000	5.000	0.917	−0.475	−0.342
TES1	4.296	4.000	1.000	5.000	0.676	1.500	−0.880
TES2	4.333	4.000	2.000	5.000	0.601	0.512	−0.510
TES3	4.275	4.000	2.000	5.000	0.655	0.789	−0.679
TAS1	4.301	4.000	1.000	5.000	0.672	1.347	−1.004
TAS3	3.906	4.000	1.000	5.000	0.751	1.920	−0.888
PS2	4.002	4.000	1.000	5.000	0.811	1.697	−1.024
PS3	3.930	4.000	1.000	5.000	0.879	0.558	−0.814
STRESS1	3.497	4.000	1.000	5.000	1.058	−0.747	−0.284
STRES2	3.271	3.000	1.000	5.000	1.094	−0.910	−0.007
STRES3	2.793	3.000	1.000	5.000	1.015	−0.234	0.467

**Table 3:** Data for testing the measurement model and collinearity problems.

Latens Construct	Indicators	Loadings	CA	RHO-A	CR	AVE	VIF
Interest in learning	INT1	0.812	0.789	0.794	0.864	0.614	1.708
	INT2	0.779					1.605
	INT3	0.824					1.761
	INT4	0.714					1.410
Student mathematics achievement	OUT1	0.703	0.764	0.772	0.850	0.586	1.363
	OUT2	0.776					1.548
	OUT3	0.803					1.548
	OUT4	0.777					1.584
Parent support	PS2	0.847	0.726	0.731	0.808	0.678	1.146
	PS3	0.799					1.146
stress	STRES2	0.911	0.829	0.832	0.899	0.747	2.665
	STRES3	0.807					1.570
	STRES1	0.873					2.325
Teacher academic support	TAS1	0.849	0.763	0.766	0.821	0.696	1.182
	TAS3	0.819					1.182
Teacher emotional support	TES1	0.872	0.885	0.886	0.929	0.813	2.075
	TES2	0.913					2.970
	TES3	0.920					3.073
Well-being	WB1	0.868	0.794	0.797	0.880	0.710	2.020
	WB2	0.874					2.037
	WB3	0.782					1.418

**Table 4:** Discriminant validity test (Fornell–Larcker).

	Interest in L	Achievements	Parent SUP	Stress	Teacher ACA SUP	Teacher EMO SUP	Well-Being
Interest in L	**0.783**						
Achievements	0.710	**0.766**					
Parent SUP	0.345	0.384	**0.823**				
Stress	−0.447	−0.396	−0.185	**0.865**			
Teacher ACA SUP	0.485	0.471	0.348	−0.254	**0.834**		
Teacher EMO SUP	0.400	0.350	0.342	−0.278	0.539	**0.902**	
Well-being	0.707	0.621	0.307	−0.427	0.397	0.336	**0.843**

**Table 5:** HTMT.

	Interest in L	Achievements	Parent SUP	Stress	Teacher ACA SUP	Teacher EMO SUP	Well-Being
Interest in L							
Achievements	0.810						
Parent SUP	0.539	0.606					
Stress	0.548	0.493	0.274				
Teacher ACA SUP	0.729	0.718	0.639	0.367			
Teacher EMO SUP	0.480	0.424	0.495	0.324	0.756		
Well-being	0.888	0.792	0.479	0.529	0.592	0.400	

**Table 6:** Coefficient determination.

	R Square	R Square Adjusted
Interest in L	0.576	0.572
Achievement	0.564	0.559
Stress	0.097	0.092
Well-being	0.297	0.291

**Table 7:** Model fit.

	Saturated Model	Estimated Model
*SRMR*	0.067	0.067
*NFI*	0.736	0.736

**Table 8:** The final results of hypotheses testing, the value of T statistics, and the *p* value.

Hypothesis		β	Sample Mean	Standard Deviation (STDEV)	T Statistics	*p* Values	Interpretation
H1	Teacher emotional support → well-being	0.076	0.074	0.051	1.478	0.140	Not supported
H2	Teacher emotional support → stress	−0.182	−0.185	0.053	3.445	0.001	Supported
H3	Teacher emotional support → achievements	−0.025	−0.028	0.037	0.676	0.499	Not supported
H4	Teacher emotional support → interest in learning	0.060	0.065	0.055	1.087	0.277	Not supported
H5	Teacher academic support → well-being	0.225	0.227	0.050	4.519	0.000	Supported
H6	Teacher academic support → stress	−0.129	−0.126	0.062	2.066	0.039	Supported
H7	Teacher academic support → achievements	0.132	0.134	0.042	3.151	0.002	Supported
H8	Teacher academic support → interest in learning	0.180	0.175	0.068	2.633	0.008	Supported
H9	Parent support → well-being	0.143	0.144	0.044	3.224	0.001	Supported
H10	Parent support → stress	−0.078	−0.079	0.054	1.438	0.151	Not supported
H11	Parent support → achievements	0.120	0.120	0.039	3.111	0.002	Supported
H12	Parent support → interest in learning	0.072	0.071	0.030	2.401	0.016	Supported
H13	Stress → well-being	−0.323	−0.323	0.052	6.156	0.000	Supported
H14	Stress → achievements	−0.063	−0.062	0.034	1.858	0.043	Supported
H15	Stress → interest in learning	−0.145	−0.145	0.034	4.243	0.000	Supported
H16	Interest in learning → achievements	0.446	0.448	0.055	8.123	0.000	Supported
H17	Well-being → achievements	0.198	0.197	0.049	4.005	0.000	Supported
H18	Well-being → interest in learning	0.531	0.532	0.043	12.464	0.000	Supported

**Table 9:** The direct, indirect, and total effect of the research model.

Result	Factor	Direct Effect	Indirect Effect	Total Effect
Interest in learning	Parent Support	0.072	0.101	0.173
	Stress	−0.145	−0.171	−0.316
	Teacher Academic Support	0.180	0.160	0.340
	Teacher Emotional Support	0.060	0.098	0.158
	Well-being	0.531		0.531
Stress	Parent Sup	−0.078		−0.078
	Teacher Academic Support	−0.129		−0.129
	Teacher Emotional Support	−0.182		−0.182
Student mathematics achievement	Interest in Learning	0.446		0.446
	Parent Support	0.120	0.115	0.235
	Stress	−0.063	−0.205	−0.268
	Teacher Aca Sup	0.132	0.212	0.344
	Teacher Emo Sup	−0.025	0.109	0.084
	Well-being	0.198	0.237	0.435

## Data Availability

The data that support the findings of this study are available on request from the corresponding author.

## Appendix A. Questionnaire Items

**Construct**	**Indonesian Version Question-Naire**	**English Version Questionnaire**
Interest in learning mathematics	Saya suka belajar materi matematika	I like to study mathematics.
	Saya penasaran dengan ilmu matematika	I′m curious about mathematics.
	Saya suka menganalisis dan memecahkan masalah matematika	I like analyzing and solving mathematics-related problems.
	Saya suka mengaplikasikan ilmu matematika yang sudah saya pelajari di sekolah pada kehidupan sehari hari	I like to apply the mathematics that I have learned at school in my daily life.
Mathematics learning achievement	Saya mendapatkan nilai yang memuaskan pada pelajaran matematika	I get good grades in mathematics.
	Saya dapat mengerjakan Sebagian besar soal soal matematika yang diberikan oleh guru	I can finish most of the mathematics problems given by the teachers.
	Saya merasa pengetahuan saya tentang matematika meningkat secara bertahap	I feel my knowledge of mathematics is increasing gradually.
	Saya tidak mudah lupa dengan materi matematika yang sudah dipelajari di sekolah	I do not easily forget the mathematics material that I have learned at school.
Well-being	Belajar matematika membuat saya mempunyai lebih percaya diri	Learning mathematics makes me have more confidence.
	Secara keseluruhan, saya merasa percaya diri dan positif saat saya belajar matematika	Overall, I feel confident and positive when I study mathematics.
	Saya senang bermain dengan angka dan rumus matematika	I like to play with numbers and mathematics formulas.
Teachers emotional support	Guru kami mendorong kami agar tidak patah semangat saat kami mengerjakan latihan dan pr matematika	Our teachers encourage us not to get discouraged when we do mathematics exercises and homework.
	Guru kami selalu memberikan kami motivasi untuk mencapai nilai matematika yang lebih baik lagi	Our teachers always motivate us to achieve better mathematics scores.
	Guru kami selalu memberikan kami semangat saat belajar matematika	Our teachers always make us excited when learning mathematics.
Teachers academic support	Ketika saya mendapat kesulitan belajar matematika, guru membantu saya mencari jalan keluar	When I experience difficulty while learning mathematics, my teachers help me find a solution.
	Ketika saya mudah mengerti, guru memberikan saya soal matematika yang lebih sulit	When I understand the topic taught quickly, the teachers give me more complex problems to solve.
	guru saya memberikan kesempatan untuk mengerjakan soal matematika menurut ide dan cara saya sendiri	My teachers allow me to work on mathematics problems according to my ideas and ways.
Parents support	Orang tua ikut membantu saya saat belajar matematika di rumah	My parents help me when I study mathematics at home.
	orang tua saya selalu memberikan semangat dan motivasi untuk saya belajar matematika	My parents always make me excited and motivated to learn mathematics.
	Overall, orang tua saya mendorong saya untuk mendapat nilai tinggi di pelajaran matematika	Overall, my parents encouraged me to get high marks in mathematics.
Stress	Belajar matematika membuat saya lelah	Studying mathematics makes me tired.
	PR matematika membuat saya stress	Mathematics homework stresses me out.
	Saya tidak semangat belajar saat pelajaran matematika	I′m not enthusiastic about studying in mathematics class.

## References

[1] Hanham J., Lee C.B., Teo T. (2021). The influence of technology acceptance, academic self-efficacy, and gender on academic achievement through online tutoring. Comput. Educ..

[2] Bai C.-E., Chi W., Qian X. (2014). Do college entrance examination scores predict undergraduate GPAs? A tale of two universities. China Econ. Rev..

[3] Aljadani A.H., Alsolami A., Almehmadi S., Alhuwaydi A., Fathuldeen A. (2021). Epidemiology of Burnout and Its Association with Academic Performance Among Medical Students at Hail University, Saudi Arabia. Sultan Qaboos Univ. Med. J..

[4] Boateng A.A., Essel H.B., Vlachopoulos D., Johnson E.E., Okpattah V. (2022). Flipping the Classroom in Senior High School Textile Education to Enhance Students’ Learning Achievement and Self-Efficacy. Educ. Sci..

[5] Kul Ü., Çelik S., Aksu Z. (2018). The Impact of Educational Material Use on Mathematics Achievement: A Meta-Analysis. Int. J. Instr..

[6] Gunawan G., Kosim K., Lestari P.A.S. (2020). Instructional Materials for Discovery Learning with Cognitive Conflict Approach to Improve Vocational Students’ Achievement. Int. J. Instr..

[7] Huang T.-H., Liu F., Chen L.-C., Tsai C.-C. (2021). The acceptance and impact of Google Classroom integrating into a clinical pathology course for nursing students: A technology acceptance model approach. PLoS ONE.

[8] Ramirez L., Machida S.K., Kline L., Huang L. (2014). Low-Income Hispanic and Latino High School Students’ Perceptions of Parent and Peer Academic Support. Contemp. Sch. Psychol..

[9] Eutsler L. (2018). Parents’ mobile technology adoption influences on elementary children’s use. Int. J. Inf. Learn. Technol..

[10] Dityawati M.S., Wuryadi (2019). The Influence of Learning Motivation, Ability of Teachers to Teach, Parental Attention and Learning Facilities in Understanding Material of Regulatory System in Senior High School. J. Phys. Conf. Ser..

[11] Zhu J., Mok M.M.C. (2018). Predicting primary students’ self-regulated learning by their prior achievement, interest, personal best goal orientation and teacher feedback. Educ. Psychol..

[12] Sabouripour F., Roslan S., Ghiami Z., Memon M.A. (2021). Mediating Role of Self-Efficacy in the Relationship Between Optimism, Psychological Well-Being, and Resilience Among Iranian Students. Front. Psychol..

[13] Gustems-Carnicer J., Calderon C., Batalla-Flores A., Esteban-Bara F. (2019). Role of Coping Responses in the Relationship Between Perceived Stress and Psychological Well-Being in a Sample of Spanish Educational Teacher Students. Psychol. Rep..

[14] Mills R., Tomas L., Whiteford C., Lewthwaite B. (2020). Developing Middle School Students’ Interest in Learning Science and Geology Through Slowmation. Res. Sci. Educ..

[15] O’Grady G., Yew E.H.J., Goh K.P.L., Schmidt H.G. (2014). Problem-based Learning and Student Motivation: The Role of Interest in Learning and Achievement. One-Day, One-Problem: An Approach to Problem-Based Learning.

[16] Hall S.S., McGill R.M., Puttick S., Maltby J. (2022). Resilience, science, technology, engineering, and mathematics (STEM), and anger: A linguistic inquiry into the psychological processes associated with resilience in secondary school STEM learning. Br. J. Educ. Psychol..

[17] Allen D., Fraser B.J. (2007). Parent and student perceptions of classroom learning environment and its association with student outcomes. Learn. Environ. Res..

[18] Abdullah N.A., Shamsi N.A., Jenatabadi H.S., Ng B.-K., Mentri K.A.C. (2022). Factors Affecting Undergraduates’ Academic Performance during COVID-19: Fear, Stress and Teacher-Parents’ Support. Sustainability.

[19] Syamsudduha S., Ginanto D. (2017). Parental Involvement in Indonesia: A study on two Public Schools in Makassar. Advances in Social Science, Education and Humanities Research.

[20] Yulianti K., Denessen E., Droop M. (2019). Indonesian Parents’ Involvement in Their Children’s Education: A Study in Elementary Schools in Urban and Rural Java, Indonesia. Sch. Community J..

[21] Tang J., Wijaya T.T., Weinhandl R., Houghton T., Lavicza Z., Habibi A. (2022). Effects of Micro-Lectures on Junior High School Students’ Achievements and Learning Satisfaction in Mathematics Lessons. Mathematics.

[22] Wijaya T.T., Jiang P., Mailizar M., Habibi A. (2022). Predicting Factors Influencing Preservice Teachers’ Behavior Intention in the Implementation of STEM Education Using Partial Least Squares Approach. Sustainability.

[23] Wijaya T.T., Ying Z., Purnama A. (2020). Using Hawgent Dynamic Mathematic Software in Teaching Trigonometry. Int. J. Emerg. Technol. Learn..

[24] OECD (2017). PISA 2015 Assessment and Analytical Framework: Science, Reading, Mathematics, Financial Literacy and Collaborative Problem Solving Paris.

[25] Pereira J., Jianlan T., Wijaya T.T., Purnama A., Hermita N., Tamur M. (2021). Using Hawgent Mathematics Software to Help Primary School Students to Read Clocks. J. Phys. Conf. Ser..

[26] Rahmadi I.F., Lavicza Z., Kocadere S.A., Padmi R.S., Houghton T. (2021). User-generated microgames for facilitating learning in various scenarios: Perspectives and preferences for elementary school teachers. Interact. Learn. Environ..

[27] Wijaya T.T., Ying Z., Chotimah S., Bernard M., Zulfah (2020). Astuti Hawgent dynamic mathematic software as mathematics learning media for teaching quadratic functions. J. Phys. Conf. Ser..

[28] Wijaya T.T., Cao Y., Weinhandl R., Tamur M. (2022). A meta-analysis of the effects of E-books on students’ mathematics achievement. Heliyon.

[29] Mata L., Pedro I., Peixoto F.J. (2018). Parental support, student motivational orientation and achievement: The impact of emotions. Int. J. Emot. Educ..

[30] Khan R.M., Bushra M., Chohan I. (2010). Impact of Parental Support on the Academic Performance and Self Concept of the Student. J. Res. Reflect..

[31] Geng Y., Fei W., Tang Z., Wang S., Yu J., Zhang M., Zhang T. (2022). Parental care and depressive symptoms among Chinese medical students: Roles of empathy and gender. BMC Med. Educ..

[32] Ata-Aktürk A., Demircan H. (2021). Supporting Preschool Children’s STEM Learning with Parent-Involved Early Engineering Education. Day Care Early Educ..

[33] Ma L., Luo H., Xiao L. (2021). Perceived teacher support, self-concept, enjoyment and achievement in reading: A multilevel mediation model based on PISA 2018. Learn. Individ. Differ..

[34] Hidayah N.H., Pali M., Ramli M., Hanurawan F. (2016). Students’ Well-Being Assessment at School. J. Educ. Health Community Psychol..

[35] Proctor C., Linley P.A. (2013). Research, Applications, and Interventions for Children and Adolescents: A Positive Psychology Perspective.

[36] Yuill N., Martin A.F. (2016). Curling Up with a Good E-Book: Mother-Child Shared Story Reading on Screen or Paper Affects Embodied Interaction and Warmth. Front. Psychol..

[37] Cheng Y., Zhang X.M., Ye S.Y., Jin H.M., Yang X.H. (2020). Suicide in Chinese Graduate Students: A Review From 2000 to 2019. Front. Psychiatry.

[38] Zhao Y., Hong J.S., Zhao Y., Yang D. (2021). Parent–Child, Teacher–Student, and Classmate Relationships and Bullying Victimization Among Adolescents in China: Implications for School Mental Health. Sch. Ment. Health.

[39] LoCastro V. (1990). The English in Japanese university entrance examinations: A sociocultural analysis. World Engl..

[40] Prahmana R.C.I., Sutanti T., Diponegoro A.M. (2021). Mathematics anxiety and the influencing factors among junior high school students in yogyakarta, Indonesia. Croat. J. Educ..

[41] Bajaj B., Khoury B., Sengupta S. (2022). Resilience and Stress as Mediators in the Relationship of Mindfulness and Happiness. Front. Psychol..

[42] Choi Y.-J., Hyosung L. (2021). Structural Relationships among the Academic Stress, Stress Coping, Academic Self-Efficacy and Academic Burnout in Student-athletes. Sport Sci..

[43] Salanova M., Schaufeli W., Martinez I.M.M., Breso E. (2010). How obstacles and facilitators predict academic performance: The mediating role of study burnout and engagement. Anxiety Stress. Coping.

[44] Zárate-Santana Z.-J., Patino-Alonso M.-C., Sánchez-García A.-B., Galindo-Villardón P. (2021). Learning Approaches and Coping with Academic Stress for Sustainability Teaching: Connections through Canonical Correspondence Analysis. Sustainability.

[45] Vallejo-Martin M., Valle J.A., Angulo J.J.P. (2018). Perceived stress in university students: The influence of academic burnout and engagement. IJERI Int. J. Educ. Res. Innov..

[46] Selian S.N., Hutagalung F.D., Rosli N.A. (2020). Academic Stress, Coping and Social Cultural Adaptation of Psychological Well Being among Indonesian Postgraduate Students. Pertanika J. Soc. Sci. Humanit..

[47] Wijaya T.T., Tang J., Li L., Purnama A. (2021). Implementing Dynamic Mathematics Software in Calculus II for Engineering Students: Quadratic Surfaces. Software Engineering and Algorithms.

[48] Garneli V., Sotides C., Patiniotis K., Deliyannis I., Chorianopoulos K. (2019). Designing a 2D Platform Game with Mathematics Curriculum. Games and Learning Alliance, Proceedings of the 8th International Conference on Games and Learning Alliance (GALA), Athens, Greece, 27–29 November 2019.

[49] Rotgans J.I., Schmidt H.G. (2011). Situational interest and academic achievement in the active-learning classroom. Learn. Instr..

[50] Fadda D., Pellegrini M., Vivanet G., Callegher C.Z. (2021). Effects of digital games on student motivation in mathematics: A meta-analysis in K-12. J. Comput. Assist. Learn..

[51] Kastner-Hauler O., Tengler K., Sabitzer B., Lavicza Z. (2022). Combined Effects of Block-Based Programming and Physical Computing on Primary Students’ Computational Thinking Skills. Front. Psychol..

[52] Cage E., McManemy E. (2022). Burnt Out and Dropping Out: A Comparison of the Experiences of Autistic and Non-autistic Students During the COVID-19 Pandemic. Front. Psychol..

[53] Steiner-Hofbauer V., Holzinger A. (2020). How to Cope with the Challenges of Medical Education? Stress, Depression, and Coping in Undergraduate Medical Students. Acad. Psychiatry.

[54] Lin Y.-L., Huang S.W., Chang C.-C. (2019). The impacts of a marine science board game on motivation, interest, and achievement in marine science learning. J. Balt. Sci. Educ..

[55] Gopal K., Salim N.R., Ayub A.F.M. (2018). Influence of self-efficacy and attitudes towards statistics on undergraduates’ statistics engagement in a Malaysian public university. J. Phys. Conf. Ser..

[56] Shi Y., Zhang J., Yang H., Yang H.H. (2021). Effects of Interactive Whiteboard-based Instruction on Students’ Cognitive Learning Outcomes: A Meta-Analysis. Interact. Learn. Environ..

[57] Huang Y.-M., Liang T.-H., Chiu C.-H. (2013). Gender differences in the reading of e-books: Investigating children’s attitudes, reading behaviors and outcomes. Educ. Technol. Soc..

[58] Wijaya T.T., Cao Y., Weinhandl R., Yusron E., Lavicza Z. (2022). Applying the UTAUT Model to Understand Factors Affecting Micro-Lecture Usage by Mathematics Teachers in China. Mathematics.

[59] Wijaya T.T., Weinhandl R. (2022). Factors Influencing Students’ Continuous Intentions for Using Micro-Lectures in the Post-COVID-19 Period: A Modification of the UTAUT-2 Approach. Electronics.

[60] Jeoreskog K.G., Wold H.O. (1982). The ML and PLS techniques for modeling with latent variables: Historical and comparative aspects. Syst. Under Indirect. Obs..

[61] Zhou Y., Li X., Wijaya T.T. (2022). Determinants of Behavioral Intention and Use of Interactive Whiteboard by K-12 Teachers in Remote and Rural Areas. Front. Psychol..

[62] Huang C.-H. (2021). Using PLS-SEM Model to Explore the Influencing Factors of Learning Satisfaction in Blended Learning. Educ. Sci..

[63] Leow L.P., Phua L.K., Teh S.Y. (2021). Extending the social influence factor: Behavioural intention to increase the usage of information and communication technology-enhanced student-centered teaching methods. Educ. Technol. Res. Dev..

[64] Raza S.A., Khan K.A. (2021). Knowledge and innovative factors: How cloud computing improves students’ academic performance. Interact. Technol. Smart Educ..

[65] Sofwan M., Pratama R., Muhaimin M., Yusnaidar Y., Mukminin A., Habibi A. (2021). Contribution of technology innovation acceptance and organizational innovation climate on innovative teaching behavior with ICT in indonesian education. Qwerty Open Interdiscip. J. Technol. Cult. Educ..

[66] Hair J.F., Hult G.T.M., Ringle C., Sarstedt M. (2016). A Primer on Partial Least Squares Structural Equation Modeling (PLS-SEM).

[67] Hair J., Black B., Babin B., Anderson R.E., Tatham R.L. (2006). Multivariate Data Analysis.

[68] CFornell C., Larcker D.F. (1981). Evaluating Structural Equation Models with Unobservable Variables and Measurement Error. J. Mark. Res..

[69] AL-Qadri A.H., Ahmed S.A.M., Suliman M.A.E., Al-khresheh M.H., Boudouaia A., Zhao W., Zhang W. (2022). Academic expectations among international students from North-Western China: A case of technology use during and post COVID-19. Front. Psychol..

[70] Briz-Ponce L., Pereira A., Carvalho L., Juanes-Méndez J.A., García-Peñalvo F.J. (2017). Learning with mobile technologies—Students’ behavior. Comput. Hum. Behav..

[71] Maduku D.K. (2017). Understanding E-Book Continuance Intention: Empirical Evidence from E-Book Users in a Developing Country. Cyberpsychol. Behav. Soc. Netw..

[72] Dijkstra T.K. (2010). Handbook of Partial Least Squares.

[73] Henseler J., Ringle C.M., Sarstedt M. (2015). A new criterion for assessing discriminant validity in variance-based structural equation modeling. J. Acad. Mark. Sci..

[74] Lau S.C., Chow H.J., Wong S.C., Lim C.S. (2021). An empirical study of the influence of individual-related factors on undergraduates’ academic burnout: Malaysian context. J. Appl. Res. High. Educ..

[75] Fussell S.G., Truong D. (2021). Accepting virtual reality for dynamic learning: An extension of the technology acceptance model. Interact. Learn. Environ..

[76] Ain N.U., Kaur K., Waheed M. (2016). The influence of learning value on learning management system use: An extension of UTAUT2. Inf. Dev..

[77] Rabu S.N.A., Hussin H., Bervell B. (2019). QR code utilization in a large classroom: Higher education students’ initial perceptions. Educ. Inf. Technol..

[78] Mulyani E.A., Alpusari M., Putra E.D. (2021). The Effect of Learning Facilities and Family Environment on Motivation to Learn of Prospective Elementary Teacher Education on Online Learning. J. Teach. Learn. Elem. Educ..

[79] Al-Emran M., Arpaci I., Salloum S.A. (2020). An empirical examination of continuous intention to use m-learning: An integrated model. Educ. Inf. Technol..

[80] Khine M.S., Ali N., Afari E. (2017). Exploring relationships among TPACK constructs and ICT achievement among trainee teachers. Educ. Inf. Technol..

[81] Yew W.C., Kong S.M., Awang A.H., Yi G.R. (2022). Developing a Conceptual Model for the Causal Effects of Outdoor Play in Preschools Using PLS-SEM. Sustainability.

[82] Alvi I. (2021). College students’ reception of social networking tools for learning in India: An extended UTAUT model. Smart Learn. Environ..

[83] Nikou S., Aavakare M. (2021). An assessment of the interplay between literacy and digital Technology in Higher Education. Educ. Inf. Technol..

[84] Wu C.-H., Liu C.-H., Huang Y.-M. (2022). The exploration of continuous learning intention in STEAM education through attitude, motivation, and cognitive load. Int. J. STEM Educ..

[85] Cohen J. (1988). Statistical Power Analysis for the Behavioral Sciences.

[86] Larzelere R.E., Morris A.S., Harrist A.W. (2013). Authoritative Parenting: Synthesizing Nurturing and Discipline for Optimal Child Development.

[87] Paglia-Boak A., Adlaf E.M., Hamilton H.A., Beitchman J.H., Wolfe D., Mann R.E. (2012). The Mental Health and Well-Being of Ontario Students.

[88] Sang G., Liang J.-C., Chai C.S., Dong Y., Tsai C.-C. (2018). Teachers’ actual and preferred perceptions of twenty-first century learning competencies: A Chinese perspective. Asia Pac. Educ. Rev..

[89] Joubert J., Callaghan R., Engelbrecht J. (2020). Lesson study in a blended approach to support isolated teachers in teaching with technology. Zdm.

[90] Qiu C.-A., He H.-X., Chen G.-L., Xiong M.-X. (2022). Pre-service teachers’ perceptions of technological pedagogical content knowledge in mainland China: A survey of teachers of Chinese as a second language. Educ. Inf. Technol..

[91] Pascoe M.C., Hetrick S.E., Parker A.G. (2020). The impact of stress on students in secondary school and higher education. Int. J. Adolesc. Youth.

[92] Sutarto S., Sari D.P., Fathurrochman I. (2020). Teacher strategies in online learning to increase students’ interest in learning during COVID-19 pandemic. J. Konseling Dan Pendidik..

[93] Kaya M., Erdem C. (2021). Students’ Well-Being and Academic Achievement: A Meta-Analysis Study. Child Indic. Res..

[94] Mentari W.N., Syarifuddin H. (2020). Improving student engagement by mathematics learning based on contextual teaching and learning. J. Phys. Conf. Ser..

[95] Bandura A. (2001). Social Cognitive Theory: An Agentic Perspective. Annu. Rev. Psychol..

[96] Pierce R., Stacey K., Barkatsas A. (2007). A scale for monitoring students’ attitudes to learning mathematics with technology. Comput. Educ..

[97] Alalwan N., Al-Rahmi W.M., Alfarraj O., Alzahrani A., Yahaya N., Al-Rahmi A.M. (2019). Integrated Three Theories to Develop a Model of Factors Affecting Students’ Academic Performance in Higher Education. IEEE Access.

